# Expectant Management of PPROM Improves Neonatal Outcome—A Retrospective Study of 562 Patients

**DOI:** 10.3390/jcm11010214

**Published:** 2021-12-31

**Authors:** Roxana Elena Bohilțea, Ana Maria Cioca, Vlad Dima, Ioniță Ducu, Corina Grigoriu, Valentin Varlas, Florentina Furtunescu

**Affiliations:** 1 Department of Obstetrics, Gynecology and Neonatology, Filantropia Clinical Hospital, 11–13 Ion Mihalache Blv., Sector 1, 011171 Bucharest, Romania; r.bohiltea@yahoo.com (R.E.B.); vlad.dima@yahoo.com (V.D.); valentin.varlas@umfcd.ro (V.V.); 2 Department of Obstetrics and Gynecology, “Carol Davila” University of Medicine and Pharmacy, 020021 Bucharest, Romania; corigri@gmail.com; 3 Faculty of Medicine, “Carol Davila” University of Medicine and Pharmacy, 37 Dionisie Lupu, 020021 Bucharest, Romania; 4 Department of Obstetrics and Gynecology, University Emergency Hospital, 169 Splaiul Independentei Bld., Sector 5, 050098 Bucharest, Romania; 5 Department of Public Health and Management, Faculty of Medicine, “Carol Davila” University of Medicine and Pharmacy, 020021 Bucharest, Romania; pbhealth@umf.ro

**Keywords:** PPROM, premature complications, latency, expectant management, corticosteroid, neonatal outcome, prematurity, preterm birth

## Abstract

Prelabor preterm rupture of the membranes (PPROM) refers to the rupture of the membranes before 37 weeks, but also before the onset of labor. Approximately 3% of pregnancies are complicated by PPROM, which is an important cause of neonatal morbidity and mortality. The aim of the study is to demonstrate the benefit of expectant management in PPROM, compared to immediate birth, defined in our study as birth in the first 48 h. We analyzed 562 pregnancies with PPROM by gestational age groups and short-term morbidities. Material and methods: We conducted a retrospective observational analytical study, which included women with PPROM between 24 + 0 and 36 + 6 weeks. We divided the cohort into gestational age groups: group 1 gestational age (GA) between 24 and 27, group 2 GA between 28 and 31, group 3 GA between 32 and 34, group 4 GA > 35 weeks. In each group, we analyzed the benefit of the latency period (established in our study as delivery after 48 h of hospitalization) in terms of short-term neonatal complications. Result: The latency period brought a significant benefit starting with GA greater than 28 weeks; therefore, in the group with GA between 28–31, the complications were significantly statistically lower, mentioning respiratory distress syndrome (no latency 100% vs. latency 85.1%) and admission to the neonatal intensive care unit (no latency 89.7% vs. latency 70.2%). In group 3, with GA between 32–34, we reached statistical significance in terms of respiratory distress syndrome (no latency 61.8% vs. latency 39%), hypoxia (no latency 50% vs. latency 31.7%) and admission to the neonatal intensive care unit (no latency 70.2% vs. latency 47.4%). Conclusion: Expectant management of pregnancies with PPROM can bring a real benefit in terms of the incidence of complications in premature infants, but this benefit depends most on the gestational age at which the membranes ruptured and the medical conduct put into practice during the latency period.

## 1. Introduction

Prelabor preterm rupture of the membranes (PPROM) refers to the rupture of the membranes before 37 weeks but also before the onset of labor. PPROM occurs in up to 3 percent of pregnancies, and one in three premature births has this pathogenic mechanism [1]. The importance of the PPROM is given by the fact that it is the main cause of infant morbidity and mortality [2]. The most important risk factors for PPROM are: history of PPROM or prematurity, nulliparity, multiple pregnancies, BMI < 18.5 kg/m^2^, infections [3], vitamin deficiency [4], antepartum bleeding and cigarette smoking [1].

Diagnosis of PPROM is simple if the rupture is obvious, and we can notice the leakage of the amniotic fluid from the cervix. If these cannot be observed, diagnosis is based on a thorough anamnesis and some simple diagnostic tests: rapid strip tests for amniotic fluid leakage measuring the vaginal pH that becomes alkaline in the presence of amniotic fluid, analysis on the microscope slide of fluid collected from posterior vaginal fornix that crystallizes in the presence of amniotic fluid due to the high content of salts and proteins and also ultrasound evaluation [5].

Premature infants have higher mortality and morbidity rates compared to full-term infants. The risk of complications is inversely proportional to the gestational age and birth weight, but certainly, there are more other factors that influence prognosis. Complications of prematurity are divided into two major classes: short-term complications (hypothermia, respiratory abnormalities, cardiovascular abnormalities, intracranial hemorrhage, hypoglycemia, necrotizing enterocolitis, infection and retinopathy of prematurity) and long-term complications (which mainly involve neurodevelopmental outcomes such as cognitive impairment and dynamic changes over time, cerebral palsy and motor impairment) [6,7].

Nowadays, the main guidelines of premature birth are not in agreement oconcerning the protocol to be followed in the case of the PPROM regarding antibiotherapy, the use of tocolytics, benefits of magnesium sulfate administration and the repeated corticoprophilaxis.

When deciding how to manage PPROM, we need to consider a few factors, including gestational age, the availability of an appropriate level of neonatal care, the presence or absence of maternal/fetal infection, labor or abruptio placentae, fetal presentation, fetal biophysical profile and cervical status. When we suspect an intrauterine infection, abruptio placentae, fetal distress, or a high risk of cord prolapse, indicate an expeditious delivery. The expectant management is preferable for stable patients (mother and fetus) with PPROM < 34 weeks who can benefit from the antenatal corticosteroid course (ACS) that decreases the incidence of respiratory distress syndrome, intraventricular hemorrhage, necrotizing enterocolitis, sepsis and neonatal mortality by up to 50% [8], a course of prophylactic antibiotics and hospital admission throughout the period until birth. In the case of pregnancies ≥34, expectant management is only recommended if gestational parameters are suboptimal; otherwise, expeditious delivery is preferred [9].

Randomized placebo-controlled trials have shown that the administration of magnesia sulphate decreased the risk of neurological dysfunction in offspring, with the mention that this therapy is recommended only for patients with less than 34 weeks of gestation (GA) [10]. It has been demonstrated that the administration of progesterone to women with a history of premature birth can reduce the risk of recurrence of preterm birth by up to one-third [11]. The administration of drugs for acute tocolysis reduce the strength and frequency of uterine contractions and delay the labor [12]; studies recommend that this therapy should not be administered for more than 48 h [9]. Medications indicated for tocolysis are calcium channel blockers, oxytocin receptor blockers, magnesium sulphate, betamimetics and progesterone.

The aim of the study is to demonstrate the benefit of expectant management in PPROM on neonatal prognosis, analyzed by gestational age groups and short-term morbidities, compared to immediate birth, defined in our study as birth in the first 48 h of PPROM. In addition, we support the use of combined tocolytics and large spectrum antibiotherapy in the context of PPROM.

## 2. Materials and Methods

We conducted a retrospective observational analytical study in which we included pregnancies with PPROM between 24 + 0 and 36 + 6 weeks gestation who have been admitted to Bucharest Emergency University Hospital from October 2015 to November 2020. During the 5 years analyzed, out of the 1789 premature births, 562 were due to PPROM. We correlate the data of the newborn infants with the therapeutic management of the mothers. Variables analyzed to create the database can be seen in Table 1. This research is approved by Ethic Committee Comisia de Etica a SUUB (Ethics Commission of the University Emergency Hospital) (Approval Code: 73317; Approval Date: 2 December 2021).

**Figure 1:**
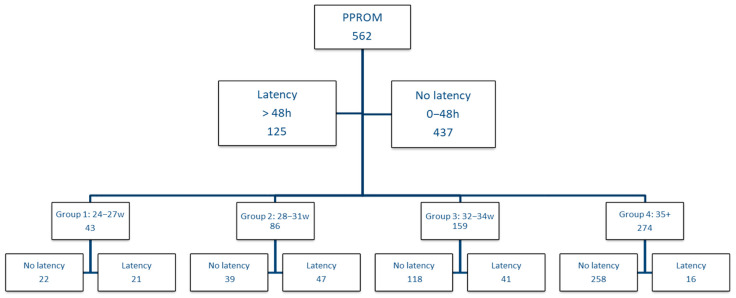
Study design.

The criteria for inclusion in the study were: ruptured membranes on presentation in the emergency room and GA between 24–36 weeks. In the case of mothers, we analyzed the duration of latency and the medication received during this time: corticotherapy, atibioprophylaxis and tocolytics. In the first phase, we aimed to analyze the duration of the latency period according to the GA, starting from the hypothesis that at a lower GA, the latency duration can be longer. Thus, we divided the entire cohort according to the duration of the latency period, respectively 0–2 days until birth vs. over 2 days until birth, and we analyzed the distribution of gestational ages in these groups. We established these cut-offs in intentions to elude the ACS administration as a bias factor. Figure 1 presents the study design.

As the influence and benefit of the latency period may be different depending on GA, we decided to perform the analysis on gestational age groups, taking into account the classification of prematurity; therefore, we divided the cohort into group 1 with GA between 24–27 weeks, group 2 between 28–31 weeks, group 3 between 32–34 weeks and in group 4 we included patients with GA over 35 weeks. Within each group, we analyzed the impact of the latency period, respectively if the birth was in the first 2 days vs. if the birth occurs after 2 days, taking into account the neonatal complications. 

The quantitative variables have been tested for normality (Kolmogorov–Smirnov test) and compared using the median test. The qualitative variables have been compared using Chi-square or Fisher test, *p* < 0.05 was considered statistically significant. 

Our approach-In most cases, we diagnose PPROM based on the clinical exam, rapid strip tests for amniotic fluid leakage and semiquantitative ultrasound assessment. The gold standard for diagnosis is the observation of amniotic fluid leakage. Our PPROM admission protocol also includes the patients with specific positive tests for ruptured membranes without clinical objectifying of the amniotic fluid leak and with the lower limit of the normal amniotic fluid level on ultrasound (classified as fissured membranes). Chorioamnionitis diagnosis is based on the presence of one of the following signs and/or paraclinical changes, not explained by other associated conditions: fetal tachycardia, fever (above 37.8 °C) present in two successive examinations at 4–6 h intervals, maternal tachycardia (over 100 bpm) present on two successive exams every 4–6 h, modified vaginal leakage and high maternal leucocytes value, over 15,000 leukocytes/mm. We perform the following laboratory test: complete blood count (CBC), C-reactive protein (CRP), ano-vaginal culture, urinalysis and urine culture. The ultrasound exam guides our management by establishing the fetal biometry and presentation, amniotic fluid residual volume, placental abnormalities. In many cases, patients present oligohydramnios (which is defined as a maximum vertical pocket (MVP) of amniotic fluid < 2 cm in depth or an amniotic fluid index (AFI) < 5 cm), that increases the risk of umbilical cord compression causing fetal distress but also decreases the latency.

Whenever possible, we opt to postpone birth by at least 48 h. The decision for expectant management is primarily taken if gestational age is over 24 weeks and under 36 weeks, and we have no signs of chorioamnionitis, abruptio placentae, fetal death, fetal non-reassuring testing or advanced labor. We administrate a course of corticotherapy which consist of 4 doses, each of 6 mg dexamethasone, 12 h interval, for lung maturation and for decreasing the risk of the other specific complications. If the birth does not occur within 7 days after the initial dose, we recommend a single repeat course after 14 days from the previous administration. As for tocolytic drugs, we always use an association of progesterone at a maximum dose of 600 mg and a calcium channel blocker or oxytocin receptor blockers or betamimetics. Since the risk of infection is high, we consider antibiotic therapy to be compulsory. We administer Ampicillin-Sulbactam (7 days) with or without methronidazole (5 days), and if the labour does not occur in the next 7 days, we replace the initial therapy with cephalosporins. Even if our guidelines recommend the administration of magnesium sulphate for fetal neuroprotection, the protocol has been only recently applied.

During the latency period, we monitor the fetus by non-stress test, cerebroplacental Doppler ratio, fetal biophysical score, and MVP twice a week. We search for leucocytosis, PCR and vaginal cultures weekly.

We recommend informing the patient about the risks and complications that may occur, such as adverse drug reactions, unfavorable evolution and complications of the premature, and we decide the conduit that is associated with as many benefits as possible.

The data collected retrospectively did not contain personal information. The study was conducted according to the guidelines of the Declaration of Helsinki.

## 3. Results

From 3 December 2015 to 4 November 2020, in our hospital, 1789 premature newborns were delivered, of whom 562 births were due to the PPROM. Of all the cases, 383 patients come from urban areas, which means about 68.2%. 307 (54.9%) of them were in the first pregnancy, 147 (26.3%) at the second pregnancy, and 62 (11.1%) at the 3rd pregnancy. Maternal age was between 14–47 years old. Table 2 present the distribution of women by age group and shows that 67% (375) are between 20–35 years old. Associated pathologies were also analyzed, and more than half (63.6%) of the analyzed women had no other health problems that would endanger their current pregnancy.

We mention that 23.3% of cervical cultures had pathogenic germs with the highest prevalence of Escherichia coli, 32.9% of patients had leukocytosis and 14.1% had CRP values above the laboratory’s reference range.

Regarding drug therapy, we have noticed that the percentage of patients who have benefited from corticotherapy is approximately equal to the percentage of women who have not received corticotherapy 53% vs. 47% (Table 3), the patients who did not benefit from corticotherapy being the ones who gave birth on the admission day. Tocolysis was administered to 43.8% of women, with the mention that 4.8% of the total tocolysis was represented by association of two drugs; 92.75% received antibiotherapy, and only six patients (1.1%) benefited from magnesium sulfate.

Regarding neonatal variables, 55.7% of the children were male, and 75% of all preterm infants had an Apgar score ≥7; 34.5% were SGA, 25.1% were LGA. Regarding the evolution of preterm infants, we have had neonatal mortality of 3%, 35.2% of newborns required intensive care, and 11.7% needed invasive mechanical ventilation. In descending order of the encountered complications, we mention: respiratory distress syndrome 41.3%, hypoxia 37.2% anemia of prematurity 21%, intraventricular hemorrhage 18.1%, hypoglycemia 17.6%, retinopathy 13.3% (Table 3).

**Figure 2:**
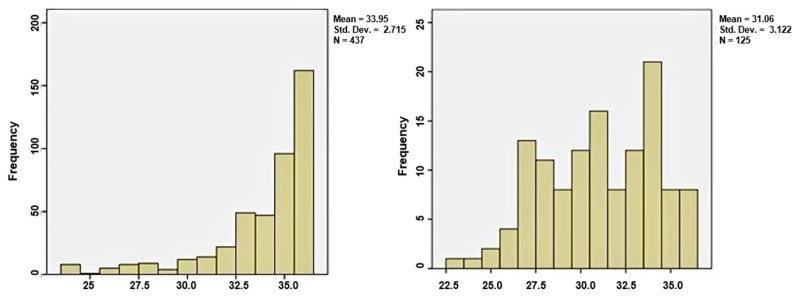
Distribution of gestational age in the two groups; (**a**) the distribution of gestational age in group 1; (**b**) the distribution of gestational age in group 2.

The first group, which delivered in the first 48 h from admission, included 437 women (77.8%), and the group of latency period over two days consisted of 125 patients, representing 22.2%. The median gestational age in the first group was 35 weeks, and in group 2 was 31 weeks, sustaining the theory that at a lower GA, the latency period can be extended more, as shown in Figure 2.

We analyzed the impact of the latency period on GA groups (Figure 1), and we included all the results obtained in Table 4. 

**Figure 3:**
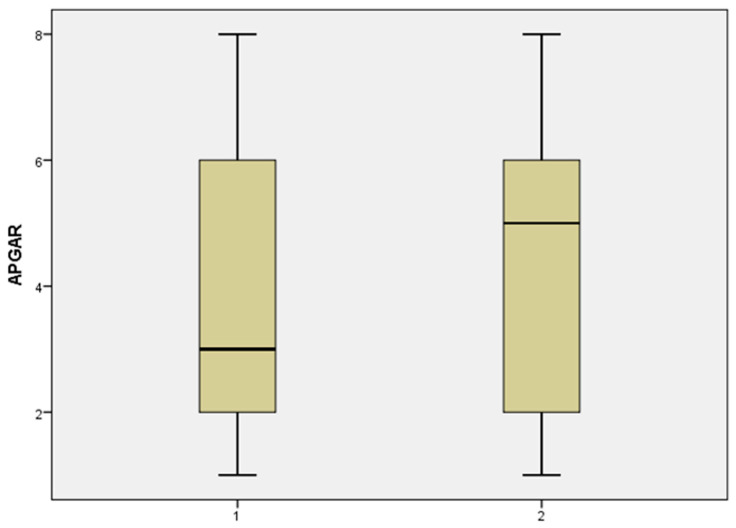
Distribution of Apgar score values in group 1 without latency vs. group 2 with latency, in case of gestational age between 24–27.

In group 1, the total number of cases included in this category was 43. The median fetal weight at birth was higher in the case of prolonged pregnancies, 900 g vs. 800 g. The Apgar score was also higher 5 vs. 3, as shown in the histogram illustrated in Figure 3. From the careful analysis of the data we obtained that the latency period has brought neonatal benefit taking into account the following complications: hypoxia (no latency (nL) 86.3% vs. latency (L) 80.9%), convulsions (nL 13.6% vs. L 9.5%), pulmonary hemorrhage (nL 36.3% vs. L 14.2%), sepsis (nL 22.7% vs. L 14.2%). However, these extreme premature babies born after 48 h had an expected bad outcome regarding intracerebral hemorrhage, which was present in 77.2% of the nL group vs. 85.7% of the L group, also hypocalcemia was four times more frequent in the latency group, retinopathy affected 50% vs. 76% of the nL and L group, anemia was 1.5 more frequent in the latency cohort (*p* < 0.05). For the patients with GA between 24 and 27 weeks, it seems that the expected management would not bring significant benefits, however in the no latency group, of the 22 newborns, 9 died (40.9%) vs. 3 (14.2%) from the latency group, which certainly is a strong argument in favor of the expectation management.

In the gestational age group between 28–31 weeks, things started to become clear in favor of expectant management; therefore, as observed in Table 4, most neonatal complications occur with a decreased incidence within the cohort with latency period. Statistical significance was achieved in the case of respiratory distress syndrome (nL 100% vs. L 85.1%, *p* = 0.015) and in the case of admission to the neonatal intensive care unit (nL 89.7% vs. L 70.2%, *p* = 0.034). Although in the no latency group, 1 out of 39 vs. 3 out of 47 newborns died in the case of the latency group, we consider that the latency period brings a proven benefit in the case of PPROM between 28 and 31 weeks.

As the gestational age increases, the benefits of latency are considerably more visible, so that in the group with GA between 32 and 34 weeks of age, it is observed that newborns from the latency group begin to have no complications. We reached statistical significance in terms of respiratory distress syndrome (nL 61.8% vs. L 39%, *p* = 0.010), hypoxia (nL 50% vs. L 31.7%, *p* = 0.047) and in the case of admission to the neonatal intensive care unit (nL 70.2% vs. L 47.4%, *p* = 0.002). In the latent group, no newborn had necrotizing enterocolitis, convulsions, acidosis, pulmonary hemorrhage, sepsis and, most importantly, none died. 

In group 4, in which the GA was over 35 weeks, neonatal complications decreased considerably, regardless of management, but with the maintenance of the differences between the group without latency period and the group with latency period, in favor of the expectant management. Although we had only 16 patients in the latency group vs. 258 in the no latency group, only 1 of them had respiratory distress syndrome, and 2 had hypocalcemia. There were no patients in the latency group admitted to the intensive care unit, who intubated or died.

## 4. Discussion

At this time, there is no standard protocol in the case of PPROM. The American College of Obstetricians and Gynecologists (ACOG), American College of Nurse-Midwives (ACNM), Royal College of Obstetricians and Gynaecologists (RCOG), World Health Organization (WHO) and Society of Obstetricians and Gynaecologists of Canada (SOGC) mention the use of corticosteroid therapy for lung maturation [13,14,15,16,17]. ACOG recommends the use of corticotherapy for women between 24–33 + 6 GA who have ruptured membranes and are at risk of delivery in the next 7 days. If the latency period is >7 days, it is recommended to repeat the dose of ACS only if 14 days have passed since the last administration. For women with GA > 34 but <36, ACOG recommends a single betamethasone course [13,16]. RCOG recommends the use of corticosteroid therapy for women with GA between 24–34 weeks of gestation [16,17], while ACNM sustain that ACS should be administered if delivery is likely within the next 7 days [14]. Although there is consensus on the benefits of antibiotic therapy, a common antibiotic regimen is not yet established in PPROM. Table 5 show the main recommendations for antibiotic therapy. The combination of amoxicillin/clavulanic acid is recommended to be avoided as it increases the risk of necrotizing enterocolitis in premature infants. ACOG, RCOG and WHO do not support the use of tocolytic therapy in the PPROM protocol because it does not bring significant benefits [15,16,17]. Instead, SOGC recommends the use of tocolytics [16]. At this time, in the literature, there is no sustained opinion on this topic; there are studies that claim that the use of tocolytics would increase the risk of chorioamnionitis [16], but our experience does not support this statement in conditions of the association of aggressive tocolysis with antibioprophylaxis. The recommendation to use magnesium sulphate for neuroprotection and to reduce the risk of cerebral palsy is mentioned by ACOG [16,17], National Institute for Health and Care Excellence (NICE) and WHO; NICE recommends 4 g IV bolus over 15 min, followed by an IV infusion of 1 g/h until birth or for 24 h for women with GA between 24 + 0 and 29 + 6 and should be taken in consideration for women with GA between 30 + 0 and 33 + 6 [18].

Melamed et al. conducted a retrospective study, which demonstrated that the latency period in cases with uncomplicated PPROM did not bring significant benefits compared to cases of premature birth by other causes. The study concluded that neonates in the uncomplicated PPROM group had an increased risk of adverse composite outcome (53.7% vs. 42.0%; *p* < 0.001), higher mortality (1.6% vs. 0.0%; *p* < 0.001), and respiratory morbidity (32.8% vs. 26.4%; *p* < 0.006), but nevertheless they had lower infectious risks (infectious morbidity was 3.5% vs. 4.9%), due to the antibiotic therapy [19]. We believe that one of the key points of the benefit of the latency period is precisely the continuous therapy with antibiotics that reduce the risk of infection.

A randomized trial of ORACLE I on 4826 women with PPROM compared patients receiving 250 mg erythromycin antibiotic treatment with women receiving 325 mg co-amoxiclav, both or placebo. They reached the following conclusions: newborns from women who received treatment with erythromycin vs. placebo had better evolutions, consisted in lower exogenous surfactant need (12.8% vs. 16.3%, *p* = 0.02), lower oxygen dependence at 28 days of age or older (6.9% vs. 8.9%, *p* = 0.03), a decreased positive blood culture rate (5.3% vs. 7.4%, *p* = 0.04), and lower incidence of abnormal cerebral ultrasonography (3.0% vs. 4.6%, *p* = 0.04). The co-amoxiclav group had a higher rate of necrotizing enterocolitis than placebo and had no statistically significant benefits [20]. In our study, 92.75% of the patients received antibioprophilaxis. Our national protocol supports the administration of antibiotics in all the cases with rupture of the membranes occurred more than 12 h ago; in addition, in our experience, we continue to use antibioprophilaxis until birth, with the change of the antibiotic spectrum of the antibiotic combination at 7–10 days, using the following classes of antibiotics: beta-lactamines, ampicillin with sulbactam, metronidazole and gentamicin in 5-day courses, new generation macrolides, cephalosporins and imipenem. It is important mention that antibiotic treatment is accompanied by probiotic treatment, and in the case of diarrhea we test for Clostridium.

In a meta-analysis conducted by Vidaeff et al., the neonatal benefit of corticosteroid therapy in case of membrane rupture vs. no ACS is supported (neonatal death 0.58 vs. 0.80; respiratory distress syndrome 0.67 vs. 0.82; intraventricular hemorrhage 0.47 vs. 0.79) [21,22]. Although the role of this therapy seems indisputable, some studies claim that the use of corticosteroid therapy in PPROM would increase the risk of postpartum endometriosis [21,23,24]. We believe that in the case of any therapeutic decision, we must weigh the risk vs. benefit, and in this case, we recommend the use of corticosteroid therapy as prevention for fetal respiratory distress. However, the patient must be informed of all adverse effects that may occur whatever therapeutic behavior is chosen.

In another meta-analysis, Magann et al. included over 1900 women. The authors clearly demonstrate the benefit of the corticosteroids compared to the control group in reducing the perinatal risk of intraventricular hemorrhage (IVH), grade III and IV (relative risk (RR) = 0.49, 95% confidence interval (CI) 0.25–0.96), IVH grade I-IV (RR 0.52, 95% CI 0.37–0.72) and respiratory distress syndrome (RR 0.81, 95% CI 0.67–0.98), without any significant differences in the risk of necrotizing enterocolitis, neonatal sepsis, Apgar score < 7 to 5 min, perinatal/neonatal mortality and maternal chorioamnionitis [25]. 

Mackeen et al. reported that tocolytic therapy, compared with no tocolytic therapy, was not associated with a significant effect on perinatal mortality in women with PPROM (RR 1.67; 95% CI 0.85 to 3.29) but was associated with a longer latency (mean difference (MD) 73.12 h; 95% CI 20.21 to 126.03) and fewer births within 48 h (average RR 0.55; 95% CI 0.32 to 0.95). A weaknesses of this therapy included an increase in Apgar <7 to 5 min (RR 6.05; 95% CI 1.65 to 22.23) and an increase in neonatal cases of mechanical ventilation (RR 2.46; 95% CI 1.14 to 5.34) [26]. In eligible cases, we support tocolytic therapy in combination with corticotherapy, the role of tocolysis being given by prolonging the PPROM pregnancy by at least 48 h so that we can obtain the expected results of corticosteroids on fetal lung maturation. Moreover, our study results support the benefits of prolonged pregnancy when the higher risk of chorioamnionitis is mitigated by the continued administration of antibiotics during the latency period. 

A retrospective cohort study by Wolfensberger et al. looked at the effects of long-term tocolytic therapy (>48 h) vs. a non-treated group. It concluded that tocolytics administered for more than 48 h lead to an increased risk of amniotic infection syndrome (13.9 vs. 4.3%) [27]. Illustrative in this way, in one of our cases, a 45 years old pregnant woman with twin dichorionic pregnancy obtained by in-vitro fertilization who suffered PPROM of one of the amniotic sac at 22 GA; we to opt for an expectant management using aggressive tocolytic therapy with betamimetics, calcium channel blockers, progesterone in combination with corticosteroid therapy and alternative antibiotic regimes. We obtained a prolongation of the pregnancy with 4 weeks. We delivered the first baby with hydrocephaly caused by oligoamnios, both placenta were left in utero, and the second fetus was born at 28 GA with an uneventful outcome [28].

Horton et al. performed a secondary analysis of a randomized controlled trial to observe the effects of magnesium sulfate on PPROM. The total group of 1259 women was divided into a cohort that received magnesium sulfate and a placebo group, and no significant differences were observed between the two groups in case of delivery < 48 h (22.2 and 20.7%, *p* = 0.51), delivery < 7 days (55.4 and 51.4%, *p* = 0.16). The benefit of magnesium sulphate turns out to be a significant decrease in the rate of IVH (grades 3 or 4) compared with placebo (0.7 vs. 2.2%; odds ratio [OR], 0.31; 95% CI 0.10–0.96) [29]. Romanian guidelines recommend magnesium sulphate in case of spontaneous rupture of membranes < 32 GA, being included in tocolytic medication [30].

The importance of the latency period regarding the delivery is also pointed out by the maternal evolution. A retrospective cohort study conducted by Bendix et al. reported that major maternal complications occurred in 59% of PPROMs between 22–27 GA and 9% in PPROMs of 28–33 GA [31].

A randomized clinical trial conducted by Abdali et al. on a total of 120 patients with PPROM at pregnancy ages between 26 and 32 weeks demonstrates the benefit of progesterone suppositories (400 mg per night) administered until delivery or until 34 GA, compared to a placebo group. The median latent phase was significantly higher in the progesterone group 8.5 days vs. 5 days in the control group in the 28–30th weeks of gestation; the mean birth-weight was significantly higher in the intervention group [32]. 

## 5. Conclusions

Expectant management of pregnancies with PPROM can bring a real benefit in terms of the incidence of short-term complications in premature infants. However, we believe that this depends on several important factors, including the gestational age at which the rupture of membranes occurred, as we have shown that latency is higher in young gestational ages. Expectant management brings obvious benefits, especially in the case of GA between 28 and 34. Another factor that influences the benefit of the latency period consists in the medical conduct that is put into practice during this time, and in this case, there is currently no standardized protocol. The benefits of corticosteroid therapy are undeniable, but they can only be achieved if it is associated with proper tocolytic therapy and continuous antibiotic therapy from rupture of membranes to birth, with the change of the antibiotic spectrum.

We consider that pregnancies complicated with PPROM are currently a topic of interest and that more studies need to be conducted on this topic.

## 6. Limitations of the Study

Although the anamnesis was carried out with meticulousness, the time elapsed from the spontaneous rupture of the membranes to the presentation in the emergency room cannot be certified for sure, this being a subjective aspect but with considerable importance.

The protocol for the administration of tocolytics and antibiotics during pregnancy is not standardized in Romania, the authors opting for the combination of orally administered progesterone in case of PPROM, corroborated with 72 h of oxytocin receptor inhibitors, or calcium channel blockers 20 mg at 12 h, or betamimetics, or indomethacin 200 mg/day between 24–28 weeks.

A special mention has to be made regarding the resistance spectrum to usual antibiotics of common germs in states where antibiotherapy is not severely restricted.

## Figures and Tables

**Table 1:** Variables analyzed.

Neonatal Data	Maternal Data
Gender	Age
Apgar score	Gestational age
AGA/SGA/LGA	Environment
Necrotizing enterocolitis	(urban/rural)
Respiratory distress syndrome	Date of admission
Hypoxia	Hours after rupture of membranes at admission
Convulsions	Date of birth
Acidosis	Number of births in history
Intracerebral hemorrhage	Associated pathologies
Pulmonary hemorrhage	Leucocytosis (yes/no)
Hypoglycemia	Increased values of CRP (yes/no)
Hypocalcemia	Cervical culture
Retinopathy	Corticotherapy (yes/no)
Anemia	Progesteron therapy(yes/no)
Sepsis	Antibiotics
Admission to the neonatal intensive care unit	Tocolysis
Invasive mechanical ventilation	Magnesium sulfate
Neonatal death	Gestational age

**Table 2:** Distribution of maternal age in the analyzed cohort.

Maternal Age	No.	%
<20 years old	24	4%
20–34 years old	375	67%
35+ years old	163	29%
Total	562	100%

**Table 3:** The incidents of the main maternal and neonate analyzed variables.

Characteristic	No. (%)
**Maternal**	
Associated pathologies	204 (36.4)
Leucocytosis	185 (32.9)
PCR	79 (14.1)
ACS	298 (53.0)
Tocolysis	6 (1.10)
Antibiotherapy	521 (92.7)
Magnesium Sulfate	6 (1.10)
**Infant**	
Male sex	313 (55.7)
Apgar > 7	426 (75.7)
SGA	194 (34.5)
Necrotizing enterocolitis	14 (2.5)
Respiratory distress syndrome	231 (42.7)
Hypoxia	240 (42.7)
Convulsions	10 (1.8)
Acidosis	22 (3.9)
Intracerebral hemorrhage	111 (19.7)
Pulmonary hemorrhage	16 (2.8)
Hypoglycemia	99 (17.6)
Hypocalcemia	68 (12.1)
Retinopathy	75 (13.3)
Anemia	118 (21.0)
Sepsis	7 (1.2)
Admission to the neonatal intensive care unit	198 (35.2)
Invasive mechanical ventilation	66 (11.7)
Death	17 (3)

**Table 4:** Analysis of neonatal complications by gestational age groups, taking into account the existence or not of the latency period.

	Group 1 GA 24–27		Group 2 GA 28–31		Group 3 GA 32–34		Group 4 GA 35+	
	No latency	Latency	No latency	Latency	No latency	Latency	No latency	Latency
Total	22	21	39	47	118	41	258	16
Median Apgar	3	5	6	6	8	8	9	8.5
Necrotizing enterocolitis	3 (13.6%)	3 (14.2%)	3 (7.6%)	2 (4.2%)	2 (1.6%)	0	1 (0.3%)	0
Respiratory distress syndrome	19 (86.3%)	19 (90.4%)	39 (100%)	40 (85.1%)	73 (61.8%)	16 (39%)	24 (9.3%)	1 (6.2%)
Hypoxia	19 (86.3%)	17 (80.9%)	34 (87.1%)	35 (74.4%)	59 (50%)	13 (31.7%)	32 (12.4%)	0
Convulsions	3 (13.6%)	2 (9.5%)	3 (7.6%)	0	2 (1.6%)	0	0	0
Acidosis	1 (4.5%)	2 (9.5%)	3 (7.6%)	0	10 (8.4%)	0	6 (2.3%)	0
Intracerebral hemorrhage	17 (77.2%)	18 (85.7%)	21 (53.8%)	21 (44.6%)	23 (19.4%)	5 (12.1%)	6 (2.3%)	0
Pulmonary hemorrhage	8 (36.3%)	3 (14.2%)	1 (2.5%)	1 (2.1%)	1 (0.8%)	0	2 (0.7%)	0
Hypoglycemia	2 (9%)	1 (4.7%)	6 (15.3%)	9 (19.1%)	26 (22%)	13 (31.7%)	40 (15.5%)	2 (12.5%)
Hypocalcemia	1 (4.5%)	4 (19%)	7 (17.9%)	2 (4.2%)	20 (16.9%)	6 (14.6%)	28 (10.8%)	0
Retinopathy	11 (50%)	16 (76%)	13 (33.3%)	23 (48.9%)	9 (7.6%)	1 (2.4%)	2 (0.7%)	0
Anemia	13 (59%)	19 (90%)	27 (69.2%)	28 (59.5%)	22 (18.6%)	3 (7.3%)	6 (2.3%)	0
Sepsis	5 (22.7%)	3 (14.2%)	1 (2.5%)	1 (2.1%)	4 (3.3%)	0	8 (3.1%)	0
Admission to the neonatal intensive care unit	21 (95.4%)	20 (95.2%)	35 (89.7%)	33 (70.2%)	56 (47.4%)	8 (19.5%)	25 (9.6%)	0
Invasive mechanical ventilation	14 (63.6%)	12 (57.1%)	14 (35.8%)	10 (21.2%)	11 (9.3%)	0	5 (1.9%)	0
Death	9 (40.9%)	3 (14.2%)	1 (2.5%)	3 (6.3%)	2 (1.6%)	0	3 (1.1%)	0

**Table 5:** The main recommendations of the guidelines regarding antibiotic therapy.

Guideline	Recommended Antibiotic Therapy
WHO [15]	Erythromycin
RCOG [16,18]	Erythromycin for 10 days
SCOG [16]	Ampicillin 2 g iv/6 h + erythromycin 250 mg iv/6 h for 48 h Followed by amoxicillin 250 mg po/8 h + Erythromycin 333 mg po/8 h for 5 days or Erythromycin 250 mg po every 6 h for 10 days
ACOG [16,17]	Intravenous (IV) Ampicillin and Erythromycin for 7 days followed by oral (po) Amoxicillin and Erythromycin

## Data Availability

The datasets used and analyzed during the current study are available from the corresponding author upon reasonable request.
